# Correspondence

**Published:** 1891-07

**Authors:** 


### Correspondence.

*Dr. H. U. Williams presents a Synopsis of the last four Park-Mütter Lectures given before the Philadelphia College of Physicians.*

*Editors Buffalo Medical and Surgical Journal:*

THE last four of the ten Mütter lectures were delivered by Dr. Roswell Park on the evenings of May 12th to 15th. As in the first six lectures, of which a synopsis was given in the issue of your Journal for February last, the time was devoted to topics relating to surgical bacteriology.

After describing the most important of the surgical infectious diseases, not taken up previously, a study was made of mixed and secondary infections. Mixed infection was defined as the introduction into the organism of two different infectious elements at about the same time. Thus, discharge of pus in gonorrhea depended not only on inoculation with the specific gonococcus, but also on staphylococci. In secondary infection, although two separate infectious elements were present, a certain sequence of events was observed; and an appreciable time, from a few hours to several months, intervened between the inoculations. Example of this was found in the suppuration of a gumma, secondary infection with pyogenic cocci occurring after primary infection with the microörganisms of syphilis. Even tertiary infection was shown to be possible, for instance, where dental caries was the primary process, thus permitting the entrance of the tubercle bacillus to neighboring lymphatic glands, which later suppurated on account of the admission of strictly pyogenic bacteria.

The affections considered subsequently to the study of mixed and secondary affections were treated of chiefly in their relation to those subjects. Their various surgical sequelæ were enumerated, attention being particularly drawn to the frequency of the inflammatory diseases of the bones and joints among them.

On account of the considerable number of conditions discussed, it has not been thought expedient to attempt condensing these lectures into a brief synopsis. The printed syllabus which was provided at the hall of the College of Physicians, is given below:

LECTURE VII.—MAY 12TH.

*Actinomycosis:*—History. Description of the disease, and of the fungus. Actinomycosis in man. Paths of infection. *Anthrax:* —History. Sources of infection. Bacillus anthracis : its characteristics ; intensification and diminution of its virulence. *Malignant Edema:*— Gangrenous emphysema. Description of the disease. Anatomical characteristics. Description of its bacillus ; biological peculiarities. Immunity enjoyed by certain animals. *Rauschbrand:*—Symptomatic anthrax. Strong resemblance to malignant edema ; essential differences. Description of its bacilli. *Glanders and Farcy:*—Brief reference to its infectious organism ; the bacillus mallei. Difficulties of diagnosis.

LECTURE VIII.—MAY 13TH.

*Tuberculosis:*—Slowness of English and American writers to properly appreciate the matter of surgical tuberculosis. Tuberculosis of lymphatic glands ; of bone ; of joints ; of tendon sheaths. Character of infectious granuloma everywhere the same. *Introduction to the study of mixed and secondary infections:*— Definition of the terms. Their evidences met with everywhere about the body. The most interesting effects, for the surgeon, are met with in the bones and joints. *Dysentery. Cholera. Hydatid cysts.*

LECTURE IX.—MAY 14TH.

*Pneumonia. Influenza. Measles. Scarlatina. Typhoid. Diphtheria. Septic angina. Mumps.*

LECTURE X.—MAY 15TH.

*Erysipelas. Lymphangitis. Variola. Cerebro-spinal meningitis. Infectious pseudo-rheumatism. Infectious endocarditis. Erythema multiforme. Tuberculosis Glanders. Anthrax. Syphilis. Gonorrhea, the Puerperal State. Other genito-urinary lesions.*

H. U. W.

---

*The Chautauqua County Medical Society—Honors to Dr. A. Waterhouse, of Jamestown—His Microscopical Researches.*

*Editors Buffalo Medical and Surgical Journal:*

AT THE last semi-annual meeting of the Chautauqua County Medical Society, the members of the Society were the guests of its President, Dr. A. Waterhouse, of Jamestown. It was to celebrate forty years of active practice. The occasion was a very pleasant one to all who had the good fortune to be present.

Among the papers of the afternoon was one by Dr. Richmond, of Fredonia, who chose for his subject, The Physician and His Microscope. In the course of the paper, the doctor took occasion to compliment Dr. Waterhouse on account of some of his achievements with the instrument, showing what a veteran he is in microscopical research; how he has made from one hundred to two hundred measurements of the red blood corpuscles of over seventy different mammalia; how the largest and smallest calibers were recorded, and the mean diameter was found by adding all the measurements together and dividing; how his collection of parasites alone will occupy an evening with the microscope, cursorily examinined,— to say nothing of his histological, pathological, and miscellaneous collections.

The privilege of entertaining our friends at our fortieth anniversary of practice will not be granted many of us. Should it be, however, it is to be hoped that we shall have a record of time as well spent.

. . . .

June, 1891.

---

THE NEW YORK PHYSICIANS' MUTUAL AID ASSOCIATION has made a splendid showing of economy and increase in membership during the past year. Its annual report merits careful examination by all the members, and others interested in the good work of the Association. Under the presidency of Dr. Daniel Lewis, this great charity may well feel proud of its record, and promises to increase its efficiency to a still greater degree. The secretary, Dr. Robert Campbell, who has kept the finances in such excellent condition, may be addressed at 1311 Broadway, care Philip Schmidt, by all who desire information in regard to the Association.

---

P. BLAKISTON, SON & CO., the Medical Publishers of Philadelphia, announce for early publication, A Hand-book of Local Therapeutics, being a practical description of all those agents used in the local treatment of disease, such as Ointments, Plasters, Powders, Lotions, Inhalations, Suppositories, Bougies, Tampons, etc., and the proper methods of preparing and applying them. The work will form a compact volume of about 400 pages, arranged in a manner to facilitate reference, and containing, besides the usual index, a complete index of diseases, that will greatly enhance its usefulness.

